# Calculating total-tract apparent digestibility is not different when adjusting or not adjusting intake for the ort nutrient composition for lactating cow diets

**DOI:** 10.3168/jdsc.2025-0903

**Published:** 2026-01-16

**Authors:** A.L. Kerwin, C.M. Ryan, G. Wittmeyer, T.R. Overton

**Affiliations:** Department of Animal Science, Cornell University, Ithaca, NY 14853

## Abstract

In a method agreement study, as part of a larger randomized controlled trial, the total-tract apparent digestibilities (TTAD) of DM, starch, and ash-corrected neutral detergent fiber (aNDFom) were calculated and compared between using the orts-adjusted diet, which accounts for the nutrient content of the TMR and orts, versus using the TMR-adjusted diet, which accounts only for the nutrient content of the TMR. Multiparous Holstein cows (n = 28) between 32 and 71 DIM were enrolled and moved to a freestall pen with Calan gates (American Calan, Northwood, NH) and fed a lactating diet high in corn silage for 10 weeks. Total-tract apparent digestibility was measured 3 times during the study, with samples collected on 2 consecutive days at each time point. Bland–Altman plots were generated and data were analyzed using SAS software (v. 9.4, SAS Institute Inc.) with a paired *t*-test to evaluate mean bias between the methods and with Pearson correlations to determine the agreement between methods and between the means and differences of the 2 methods. Lin's concordance correlation coefficient (CCC) was calculated to assess the agreement between the 2 methods. Significant correlation was found between the orts- and TMR-adjusted diets for DM, starch, and aNDFom TTAD and excellent agreement, as measured by CCC, between the methods for all nutrient TTAD. Evidence suggested that the mean difference between methods was different from 0 for DM, starch, and aNDFom TTAD; however, the mean bias was minimal. In addition, we did observe a correlation between the bias and the magnitude for DM and starch TTAD, but not for aNDFom. Overall, there was excellent agreement between methods, and the mean bias and correlation between the difference and mean TTAD values observed are minimal and likely not to be analytically significant. In conclusion, analyzing the nutrient composition of the orts may not be necessary when calculating TTAD for lactating cow diets.

## Highlights

• Total-tract apparent digestibility (TTAD) was adjusted and not adjusted for orts.
• Excellent agreement was observed between methods for DM, starch, and NDF TTAD.
• Not adjusting for orts was accurate and precise compared with orts-adjusted TTAD.
• Ort samples do not need to be collected for calculating lactating cow TTAD.

## Graphical Abstract

**Figure:**
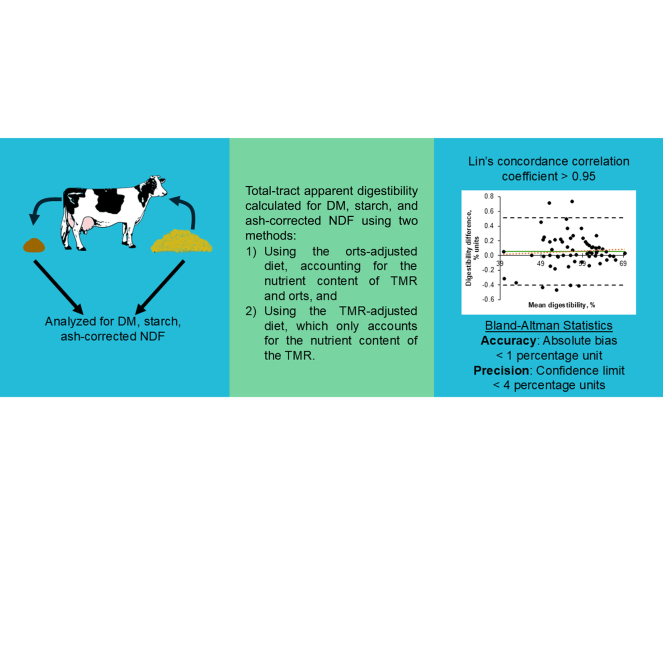
**Summary:** Total-tract apparent digestibility (TTAD) was calculated for dry matter (DM), starch, and ash-corrected neutral detergent fiber (NDF) using 2 methods; either accounting for the nutrient content of the orts or not. Lin's concordance correlation coefficient for all 3 TTAD measurements resulted in excellent agreement between methods. Bland-Altman graphs demonstrated that TTAD calculated without the ort nutrient composition was highly accurate and precise compared with TTAD when calculated with the ort nutrient composition. We conclude that ort samples do not need to be collected for calculating TTAD for lactating cow diets.

Methods for calculating total-tract apparent digestibility (**TTAD**) for nutrients varies across the literature (Maulfair et al., 2011; Ferraretto et al., 2015; Faulkner and Weiss, 2017; Daniel et al., 2020). Although total collection is regarded as the gold standard (Morris et al., 2018), facility, labor, and cost limitations prevent the use of this method. Therefore, methods used across the literature for calculating TTAD without doing total collections include collecting daily TMR samples and fecal samples at various time points across different periods of time; however, respective ort samples are not collected consistently (Daniel et al., 2020; Frizzarini et al., 2024). For calculating TTAD by total collection, it has been recommended to limit feed intake so no orts remain in order to avoid selective feeding, as selective feeding may result in overestimation of digestibility if the chemical composition of the orts is not accounted for (Van Soest, 2015). For controlled research feeding trials, limiting feed intake is often not considered when evaluating TTAD, as limiting intake would have direct effects on primary outcomes evaluated, such as DMI and milk yield. Studies that do not account for the nutrient composition of the orts are potentially biasing the TTAD results; however, depending on the number of cows enrolled in the study and TTAD sample collection protocol, analyzing the chemical composition of the ort samples for each cow could be a significant cost. We hypothesized that the calculated TTAD for DM, starch, and ash-corrected NDF (**aNDFom**) would be similar between TTAD calculation methods that either do or do not account for the chemical composition of the orts for cows fed a lactating cow diet. Therefore, the objective was to compare DM, starch, and aNDFom TTAD calculated by either using the chemical composition of the TMR and the feces or the chemical composition of the TMR, feces, and orts for lactating Holstein cows.

This manuscript was written following the GRRAS (Guidelines for Reporting Reliability and Agreement Studies) reporting guidelines (Kottner et al., 2011). In a method agreement study, samples were obtained from a randomized controlled trial in which all procedures involving animals were approved before the onset of the study by the Cornell University Institutional Animal Care and Use Committee (protocol 2023-0068). As such, this method agreement study was conducted retrospectively and a sample size calculation was not conducted for this method agreement study. Briefly, multiparous Holstein cows (n = 28) between 32 and 71 DIM were enrolled in October 2023 and moved to a freestall pen with Calan gates (American Calan, Northwood, NH) at the Cornell University Ruminant Center Dairy Research Farm in Harford, New York. Cows were fed a control lactating diet high in corn silage, typical of the Northeastern United States, during a 2-wk covariate period, before remaining on the control diet or starting a treatment diet for 8 wk. The control and treatment diets were formulated using the Cornell Net Carbohydrate and Protein System (v. 6.5.5., Cornell University) and were formulated identically, except for the corn silage used, as reported in Table 1. The corn silage used in each diet was the same hybrid, treated under the same growing and harvesting conditions, except the corn silage used in the treatment diet had a bacterial in-plant inoculant applied to the seed before planting (Seed2Rumen, Agrovive Biologicals). There were no notable nutrient composition differences between the corn silage of the diets (Table 1).

**Table 1:** Ingredient and nutrient compositions of TMR containing the noninoculated corn silage (control) or the inoculated corn silage (treatment)

Item	Control	Treatment
Ingredient, % of DM		
Noninoculated corn silage^1^	48.70	—
Inoculated corn silage^2^	—	48.70
Hay crop silage^3^	17.39	17.39
Corn grain, finely ground	13.91	13.91
Soybean meal	4.35	4.35
Grain mix^4^	15.65	15.65
Nutrient^5^		
DM	35.9 ± 2.2	36.7 ± 1.0
CP	15.3 ± 0.6	15.5 ± 0.8
ADF	18.4 ± 0.4	18.1 ± 0.7
aNDFom	29.7 ± 1.2	29.8 ± 0.4
Lignin	1.7 ± 0	1.8 ± 0.4
Starch	29.4 ± 0.5	29.5 ± 0.8
Sugar	2.4 ± 0.4	2.4 ± 0.1
NFC	43.7 ± 1.0	43.4 ± 0.6
Ether extract	4.33 ± 0.19	4.34 ± 0.01
Ash	6.98 ± 0.12	7.03 ± 0.16

1 Average corn silage composition was 28.5% DM, 8.1% CP, 63.0% soluble protein as a percentage of CP, 22.1% ADF, 35.4% aNDFom, 2.5% lignin, 34.0% starch, 4.91% ether extract, and 2.66% ash.

2 Average corn silage composition was 29.0% DM, 8.2% CP, 58.0% soluble protein as a percentage of CP, 22.0% ADF, 36.5% aNDFom, 1.9% lignin, 37.8% starch, 4.80% ether extract, and 2.83% ash.

3 Average haylage composition was 36.4% DM, 15.6% CP, 65.0% soluble protein as a percentage of CP, 29.9% ADF, 42.5% aNDFom, 2.6% lignin, 0.5% starch, 4.72% ether extract, and 8.46% ash.

4 Contained (% of DM) Amino Plus (heat-treated soybean meal, Ag Processing Inc.; 4.75%), ground soybean hulls (2.07%), corn germ meal (2.07%), blood meal (1.65%), sodium sesquicarbonate (1.34%), calcium carbonate (1.24%), Energy Booster 100 (commercial fat source, Milk Specialties Global; 1.03%), salt (0.41%), calcium sulfate (0.31%), magnesium oxide (0.31%), urea (0.21%), AminoShure-XM (commercial rumen-protected methionine, Balchem Corp.; 0.14%), selenium 0.06% (0.05%), vitamin mix (contained 30,073 kIU/kg vitamin A, 5,783 kIU/kg vitamin D, and 92,534 IU/kg vitamin E, Cargill Animal Nutrition; 0.02%), trace mineral mix (contained 160,211 mg/kg Zn, 142,105 mg/kg Mn, 23,684 mg/kg Cu, 3,526 mg/kg Co, 3,158 mg/kg I, and 197 mg/kg Fe; 0.02%), Rumensin (premix contained 26,400 g/t of monensin, Elanco Animal Health; 0.01%), vitamin E (contained 510,750 IU/kg vitamin E; 0.01%).

5 Mean ± SD of the nutrient, % of DM unless otherwise noted. Three and two 4-wk composite samples were analyzed for the control and treatment diets, respectively.

Cows were fed at ∼0700 to 0800 h and fed for ad libitum intake, targeting a 5% to 10% refusal rate. The diets were mixed in a mixer wagon and delivered via a Data Ranger (American Calan, Northwood, NH). Cows were individually fed using Calan gates, and the amount of feed delivered and refused was recorded daily. Samples of TMR were collected weekly and dried in a forced-air oven for 96 h at 48.5°C to determine DM. Dried samples were composited at 4-wk intervals and then ground to 2 mm using a Wiley mill (Arthur H. Thomas). The 4-wk composited samples of TMR were submitted for wet chemistry analysis at a commercial laboratory (Dairy One Cooperative, Ithaca, NY). The analyzed chemical composition is reported in Table 1.

Total-tract apparent digestibilities for DM, starch, and aNDFom were determined using undigested ash-corrected NDF measured at 240 h of in vitro fermentation (**uNDFom240**) as an internal marker (Potts et al., 2017; Morris et al., 2018). Total-tract apparent digestibility was measured 3 times during the study: once during the second week of the covariate period and every 4 wk during the treatment period. At each time point, samples were collected on 2 consecutive days, with the TMR samples (∼500 g of sample per diet per sample day, collected at feeding) collected the day before the fecal grab sample (∼300 g/cow per sample day, collected at ∼0530 h) and ort (∼500 g/cow per sample day, collected at ∼0530 h) sample collection. Day 1 samples from each respective sample collection were frozen at −20°C before compositing with the second-day samples. After the second-day collection, the composited TMR and ort samples were dried in a forced-air oven for 96 h at 48.5°C for determination of DM and then ground to 2 mm using a Wiley mill. Composited fecal samples were frozen at −20°C until samples were dried for 96 h at 50°C for determination of DM. Dried fecal, TMR, and ort samples were analyzed at a commercial laboratory (Dairy One, Ithaca, NY) by wet chemistry methods for residual moisture (method 930.15; AOAC International, 2006), starch (YSI Biochemistry Analyzer, Dairy One Cooperative, 2022), aNDFom (Van Soest et al., 1991; Dairy One Cooperative, 2022), and uNDFom240 (Goering and Van Soest, 1970; Dairy One Cooperative, 2022). Samples collected for TTAD calculations were further corrected for residual moisture.

Total-tract apparent digestibility was calculated 2 different ways: (1) using the orts-adjusted diet, which accounts for the nutrient content of the TMR and orts and (2) using the TMR-adjusted diet, which accounts only for the nutrient content of the TMR. Dry matter intake (kg) was calculated (1) by subtracting the amount of DM measured in the orts (ort wet weight × ort DM %) from the amount of DM fed (as-fed TMR weight × TMR DM %; orts-adjusted DMI) and (2) by subtracting the ort wet weight from the as-fed TMR weight and then multiplying by the TMR DM percentage (TMR-adjusted DMI). Similarly, intakes (kg) of starch, aNDFom, and uNDFom240 were calculated (1) by subtracting the amount of each nutrient measured in the orts (ort wet weight × ort DM % × ort nutrient concentration) from the amount of each nutrient fed (as-fed TMR weight × TMR DM % × TMR nutrient concentration; orts-adjusted nutrient intake) and (2) by multiplying the TMR-adjusted DMI by the TMR nutrient concentration (TMR-adjusted nutrient intake). Fecal output (kg) was calculated by (1) dividing the orts-adjusted uNDFom240 intake by the fecal uNDFom240 concentration (orts-adjusted fecal output) and (2) dividing the TMR-adjusted uNDFom240 intake by the uNDFom240 fecal concentration (TMR-adjusted fecal output). Fecal nutrient output (kg) was calculated by (1) multiplying the orts-adjusted fecal output by the fecal nutrient concentration (orts-adjusted nutrient fecal output) and (2) multiplying the TMR-adjusted fecal output by the fecal nutrient concentration (TMR-adjusted nutrient fecal output). Total-tract apparent digestibility for DM (%) was calculated by the following formulas: (1) (orts-adjusted DMI − orts-adjusted fecal output)/orts-adjusted DMI, referred to as the orts-adjusted DM TTAD, and (2) (TMR-adjusted DMI − TMR-adjusted fecal output)/TMR-adjusted DMI, referred to as the TMR-adjusted DM TTAD. Total-tract apparent digestibility for starch and aNDFom (%) was calculated by the following formulas: (1) (orts-adjusted nutrient intake − orts-adjusted nutrient fecal output)/orts-adjusted nutrient intake, referred to as the orts-adjusted nutrient TTAD, and (2) (TMR-adjusted nutrient intake − TMR-adjusted nutrient fecal output)/TMR-adjusted nutrient intake, referred to as the TMR-adjusted nutrient TTAD.

All statistical analyses were performed using SAS software (v. 9.4, SAS Institute Inc.). Bland–Altman plots (Bland and Altman, 1999) were generated in Microsoft Excel (Microsoft Corp.). Histograms of variables were visually assessed for normality using the UNIVARIATE procedure. The mean bias between the nutrient digestibility methods was assessed using a paired *t*-test (PROC TTEST). Pearson correlations between methods and between the means and the differences of the 2 methods were assessed using PROC CORR. Lin's concordance correlation coefficient (**CCC**) was calculated to assess the agreement between the 2 methods (Lin, 1989). Accuracy of the TMR-adjusted nutrient TTAD was evaluated by measuring the bias, and precision was evaluated by evaluating the limits of agreement (i.e., confidence limit). Acceptable agreement between methods was established a priori and set to a critical threshold of 1 and 5 percentage units for the bias and confidence limit, respectively.

We removed 1 cow during the study due to poor adaptation to the Calan gates, leaving 27 cows in the data set. In addition, all data were removed from 2 cows, from 3 cows at the first treatment period collection, and 9 cows from the second treatment period collection due to the Calan gates being compromised; therefore, the final data set included 63 observations. The averaged as-fed feed refusal rate was (mean ± SD) 9.0% ± 5.5%, the orts-adjusted DMI was 55.6 ± 6.1 kg/d, and the TMR-adjusted DMI was 55.4 ± 6.1 kg/d, across all time points. The mean (±SD) DM, starch, and aNDFom TTAD between methods were 69.7% ± 3.7% (range: 59.4% to 76.0%), 97.7% ± 1.3% (range: 92.2% to 99.4%), and 56.8% ± 6.3% (range: 39.9% to 69.4%), respectively. The mean (±SD) nutrient compositions of the TMR samples (% DM basis) collected for TTAD analysis across all time points were 36.4% ± 2.7% DM, 28.0% ± 1.0% starch, 30.3% ± 1.3% aNDFom, and 6.10% ± 0.43% uNDFom240. The mean (±SD) nutrient compositions of the ort samples (% DM basis) collected for TTAD analysis across all time points were 33.4% ± 3.0% DM, 28.4% ± 1.1% starch, 31.5% ± 2.3% aNDFom, and 7.42% ± 1.25% uNDFom240.

For DM TTAD, a significant correlation was detected between the orts- and TMR-adjusted diets (r = 0.97; *P* < 0.001; Figure 1A), and agreement was excellent between the methods (CCC [95% CI] = 0.958 [0.933, 0.974]). Evidence suggested that the bias was different from 0 (mean difference [95% CI] = −0.62 [−0.85, −0.40,]; *P* < 0.001), indicating that the TMR-adjusted TTAD was significantly less than the orts-adjusted TTAD. The confidence limit (Figure 2) was calculated to be 3.52 percentage units. In addition, we observed a negative correlation between the bias (the difference between the methods) and the magnitude (the mean of the methods; r = −0.25; *P* = 0.04; Figure 2A), indicating that the TMR-adjusted DM TTAD is more likely to be less than the orts-adjusted DM TTAD at a higher digestibility value than at a lower digestibility value.

**Figure 1:**
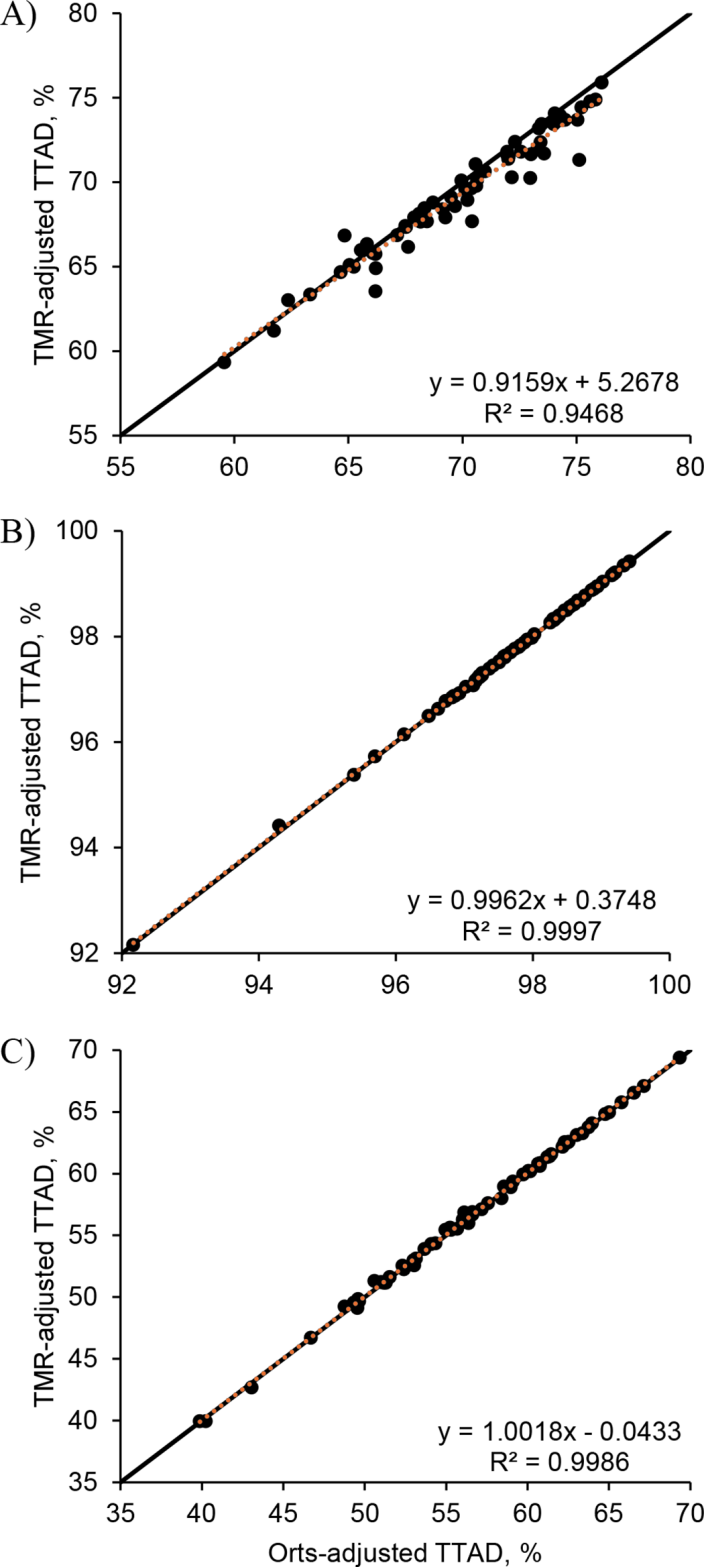
Scatterplot of the ort- and TMR-adjusted TTAD (n = 63) for (A) DM, (B) starch, and (C) aNDFom. The black solid line corresponds to the line of equality, and the linear regression line is the orange dotted line (equation displayed in the bottom right quadrant of the figure).

**Figure 2:**
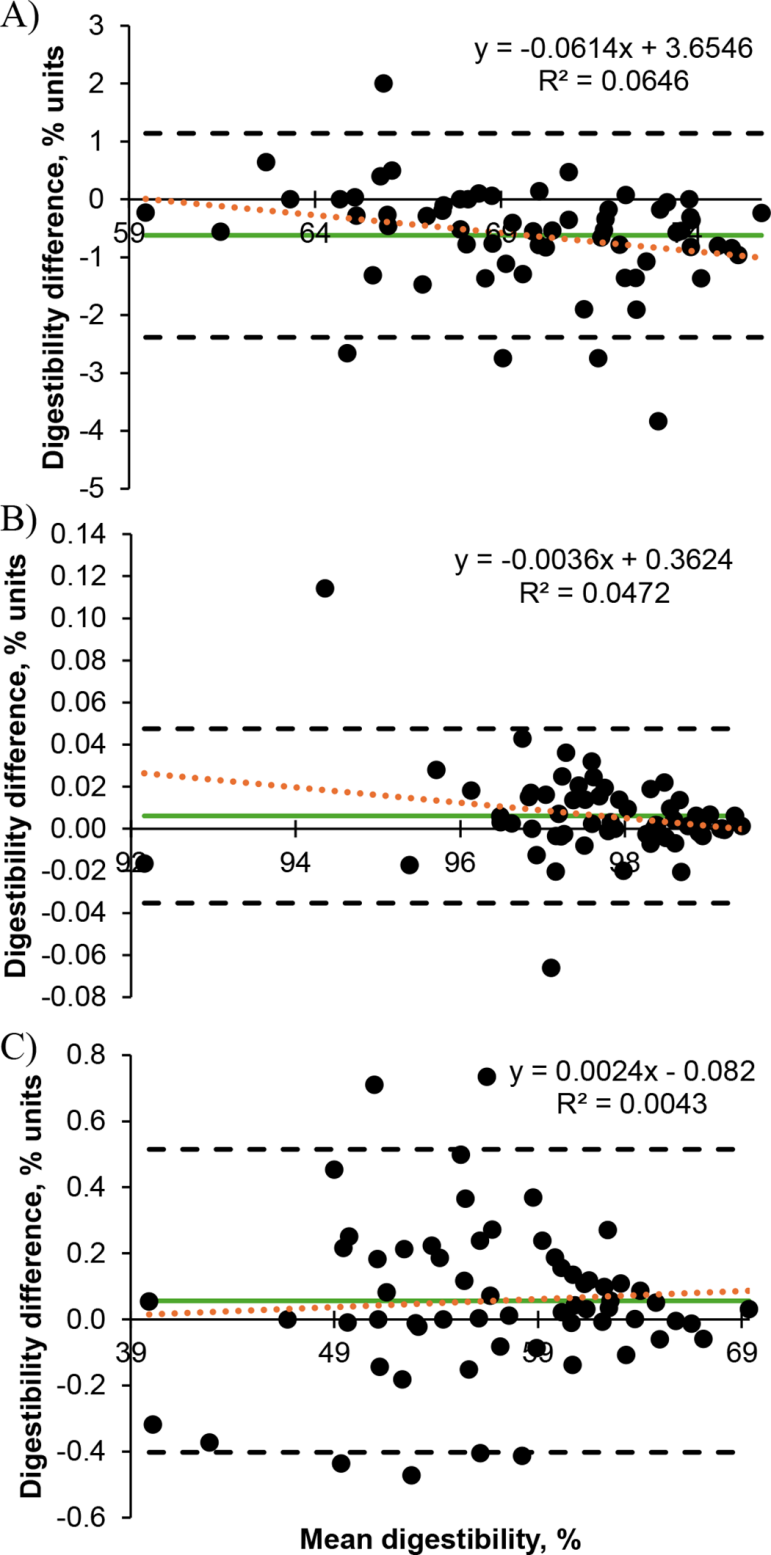
Bland–Altman plots for comparing the ort- and TMR-adjusted TTAD (n = 63) for (A) DM, (B) starch, and (C) aNDFom. The digestibility difference was calculated by subtracting the orts-adjusted nutrient TTAD from the TMR-adjusted nutrient TTAD. The mean bias is the green solid line; the linear regression line is the orange dotted line (equation displayed in the top right quadrant of the figure); and the black dashed lines are the upper and lower limits (mean bias ± 1.96 × SD).

For starch TTAD, the orts- and TMR-adjusted diets were significantly correlated (r = 0.9999; *P* < 0.001; Figure 1B), and agreement between the methods was excellent (CCC [95% CI] = 0.9998 [0.9997, 0.9999]). Similar to the DM TTAD, there was evidence that the bias was different from 0 (mean difference [95% CI] = 0.006 [0.001, 0.011]; *P* = 0.03), indicating that that TMR-adjusted TTAD was slightly greater than the orts-adjusted TTAD; however, the confidence limit (Figure 2) was calculated to be 0.08 percentage units. The bias was correlated with the magnitude (r = −0.22; *P* = 0.09; Figure 2B).

We observed a significant correlation between the orts- and TMR-adjusted diets (r = 0.9993; *P* < 0.001; Figure 1C), and agreement between the methods for aNDFom TTAD was excellent (CCC [95% CI] = 0.9993 [0.9988, 0.9996]). Evidence suggested that the bias was different from 0 (mean difference [95% CI] = 0.056 [−0.003, 0.115]; *P* = 0.06), indicating that the TMR-adjusted TTAD were greater than the orts-adjusted TTAD; however, the confidence limit range (Figure 2) was calculated to be 0.92 percentage units. We did not observe a significant correlation between the bias and the magnitude (r = 0.07; *P* = 0.61; Figure 2C), indicating the difference between the 2 methods is random as the magnitude changes.

Overall, we observed excellent agreement (CCC >0.90) between the orts- and TMR-adjusted nutrient TTAD. Even though we observed mean bias for DM and starch TTAD, the mean bias was less than our analytically acceptable difference value of 1 percentage unit. In addition, the observed confidence limits for each nutrient was less than our 5-percentage unit precision criterion, indicating that the repeatability of the TMR-adjusted diet is acceptable. We observed a negative correlation between the bias and magnitude for DM and starch TTAD; however, within the mean TTAD values observed in this study, the slope is minimal, resulting in predicted difference ranges of 1.02 and 0.03 percentage units, respectively.

We fed a lactating cow diet typical of the Northeastern and Midwestern United States; therefore, these results may not be applied to other types of diets with varying nutrient composition. Although we did not measure particle size, the corn silage and haylage were processed well. In addition, the moisture content of the TMR in this study was adequate (Table 1). Greater particle size, particularly particles on the 19-mm sieve of the Penn State Particle Separator, and greater TMR DM content have the potential to increase sorting behavior (Kononoff et al., 2003; Miller-Cushon and DeVries, 2017). Minimal sorting likely occurred in our study, resulting in minimal nutrient differences between the as-fed TMR and the orts, despite our observed higher refusal rate. For measuring TTAD, it has been recommended to limit feed intake so no orts remain to avoid selective feeding (Van Soest, 2015); however, sorting may not have occurred, in part due to cows being fed individually with the Calan feed bins. When fed individually, cows may sort throughout the day, but the feed consumed over the course of the day will be similar to the offered TMR (Maulfair and Heinrichs, 2013). These results can likely not be applied to dry cow or sortable diets. This study also assumes that the TMR was evenly mixed and distributed to all cows. If feed is not properly mixed, individual cow TMR samples should be collected and analyzed for nutrient composition, rather one TMR sample per diet. We conclude that the TMR-adjusted diet may be used for measuring DM, starch, and aNDFom TTAD for multiparous Holstein dairy cows fed a similar lactating cow diet instead of the orts-adjusted diet.
